# Pediatric Drug-Induced Sleep Endoscopy: Technique and Scoring System

**DOI:** 10.7759/cureus.10765

**Published:** 2020-10-02

**Authors:** Adrian Williamson, Samira R Ibrahim, Steven W Coutras, Michele M Carr

**Affiliations:** 1 Department of Otolaryngology, Head and Neck Surgery, West Virginia University, Morgantown, USA; 2 Department of Otolaryngology, Head and Neck Surgery, West Virginia School of Osteopathic Medicine, Lewisburg, USA; 3 Department of Otolaryngology, Head and Neck Surgery, University at Buffalo, Buffalo, USA

**Keywords:** drug induced sleep endoscopy, pediatric obstructive sleep apnea, sleep apnea surgery, dise, osa, tonsillectomy, adenoidectomy

## Abstract

Drug-induced sleep endoscopy (DISE) is an invaluable tool for identifying sites of obstruction for patients with obstructive sleep apnea (OSA). During DISE, the patient is in a state of drug-induced sleep, and a flexible laryngoscope is passed through the nose into the upper airway. Sites of obstruction are visualized and scored to guide surgical management. Currently, there is no universally accepted method of DISE analysis and scoring. This limitation in comparability impedes large-scale analysis between clinicians, institutions, and studies. In this report, we propose a standardized method of scoring and performing DISE in children with OSA. Our DISE scoring system is internally developed, consistent through the study, and addresses all levels of potential upper airway obstruction.

## Introduction

Obstructive sleep apnea (OSA) is a common childhood syndrome that can be associated with significant morbidity and is known to be detrimental to neurobehavioral, cardiovascular, endocrine, and metabolic health and development in children [1]. The prevalence of OSA is between 1% and 4%, however, it is likely under-diagnosed and under-treated [2,3]. The diagnostic gold standard for OSA is overnight polysomnography (PSG); however, this does not provide anatomic information related to the cause of airway obstruction.

Drug-induced sleep endoscopy (DISE) in children is a useful tool for identifying sources of obstruction and planning future surgical intervention. During this procedure, the patient is in a state of drug-induced sleep, and a flexible laryngoscope is passed through the nose into the upper airway. Sites of obstruction are visualized and documented, and this information is used to guide surgical management. In general, DISE demonstrates statistical promise in terms of safety, validity, test-retest reliability, and inter/intra-rater reliability [4-7]. The purposed indications for DISE in children include documented persistent OSA after tonsillectomy and adenoidectomy, high risk for persistent OSA after tonsillectomy and adenoidectomy, significant sleep-disordered breathing (SDB) or OSA in patients with small tonsils and adenoids, concern for occult or sleep-state dependent laryngomalacia, and to confirm the level of obstruction prior to placement of hypoglossal nerve stimulator [5].

There are several published scoring systems, however, none are used universally. The lack of consensus prevents objective outcome analysis between clinicians, institutions, and studies [8]. This limitation in comparability is important to consider as it may impede large-scale analysis such as systematic reviews and meta-analyses of refractory OSA patients. Commonly used DISE scoring systems differ in how many sites of obstruction are evaluated, description of airway narrowing, and obstruction configurations [5,9]. In this report, we propose a standardized method of scoring and performing DISE in children with refractory OSA. This comprehensive and easy to follow method takes into account all potential sites of obstruction, degree of narrowing, and obstruction configuration.

## Technical report

DISE is performed in the operating room with a pediatric anesthesia team that provides the necessary sedation. Following standard protocol for pediatric patients, anesthesia is first induced using an inhaled anesthetic agent allowing for intravenous access to be obtained. The inhalation agent is then discontinued and a propofol infusion dosed appropriately for the child’s age and weight is used for the remainder of the procedure. The patients are monitored closely while placed in supine position with the chin in neutral position.

**Figure 1:**
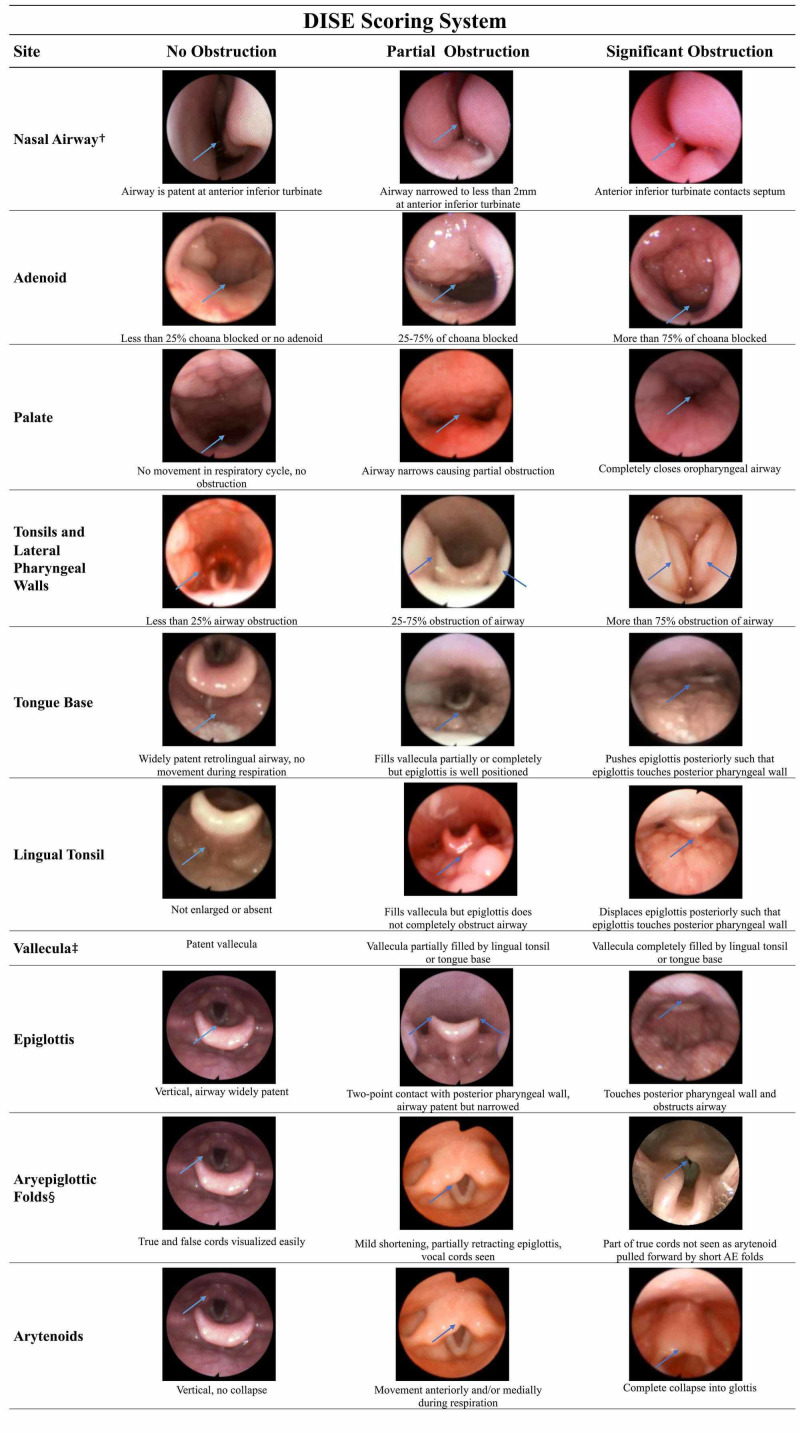
Drug-induced sleep endoscopy (DISE) scoring system This figure depicts the scoring system used with overlying picture examples. †Only left turbinate example pictures are provided, however, in practice the right and left nasal airway documented separately; ‡For vallecula example pictures, see tongue base and lingual tonsil rows. §Aryepiglottic folds were visualized with and without chin lift during DISE.

DISE is initiated when audible or palpable snoring is noted. The flexible fiberoptic laryngoscope is passed into the nose bilaterally. The flexible laryngoscope is advanced into the nasopharynx, followed by the oropharynx, and ends at the level of the supraglottic larynx. The airway patency is evaluated throughout the exam. The DISE procedures are recorded in video form and still images are taken during the procedure for later review and documentation (Figure 1). The DISE procedure is documented using a uniform template and scoring system (Figure 1). The scoring system records the operator’s evaluation of the bilateral nasal airway, adenoid, retropalatal airway, oropharyngeal airway, retrolingual airway, and laryngeal airway separately. Obstruction at each site or by the specified structure is broadly categorized as non-obstructive, partially obstructive, or significantly obstructive (Figure 1). The completed template results in a narrative record of the DISE findings for that case.

## Discussion

The scope of DISE research is limited by lack of consistent quantitative data and range of methodology used among institutions and otolaryngologists. The adoption of a standard method and scoring system for pediatric DISE would help remedy this constraint on DISE research. The most obvious limitation is the lack of consistent scoring systems among studies and institutions. Six different scoring systems (Bachar, Boudewns, Chan, Fishman, Sleep Endoscopy Rating Scale (SERS), Velum, Oropharynx and lateral pharyngeal walls, Tongue bass and Epiglottis (VOTE)) have been used to report pediatric DISE findings (Table 1) [10-15]. The goal of all these scoring systems is to concisely and consistently communicate findings during DISE in a manner that can be quantified and studied. However, these systems are each unique with regard to the anatomic sites included in the system and in their manner of quantifying and characterizing airway.

**Table 1:** Drug-induced sleep endoscopy (DISE) scoring systems used in children This table summarizes the published scorings systems used in pediatric DISE. SERS = Sleep Endoscopy Rating Scale; VOTE = Velum, Oropharynx and lateral pharyngeal walls, Tongue bass and Epiglottis; OSA = Obstructive sleep apnea.

DISE scoring systems used in children.			
Scoring System	Sites Evaluated	Quantification of obstruction	Additional comments
Bachar et al. 2012 [10]	1. Nasal airway and Nasopharynx	Score of 1 or 2 given for partial or complete obstruction respectively	This grading system converts to a NTPHL staging system. Sites without obstruction are not included in the staging for that patient.
	2. Palate and tonsils		
	3. Tongue base		
	4. Hypopharynx		
	5. Larynx		
Boudewyns et al. 2014 [11]	1. Adenoid	Score of 0 for no obstruction, 1 for less than 50% obstruction, 2 for between 50-75% obstruction, and 3 for greater than 75% obstruction	This system includes a general impression of hypotonia as present or absent.
	2. Palate	Score of 0 for no collapse, 1 for collapse present	
	2. Tonsils and oropharynx	Score of 0 for no obstruction, 1 for less than 50% obstruction, 2 for between 50-90% obstruction, and 3 for tonsils that touch at midline.	
	3. Tongue base	Score of 0 for no obstruction, 1 for partial obstruction, 2 for complete obstruction	
	4. Epiglottis	Score of 0 for no collapse, 1 for collapse present	
	5. Larynx	Laryngomalaica is noted to be absent (score of 0) or present (score of 1)	
Chan et al. 2014 [12]	1. Adenoid	Score of 0 for no obstruction, 1 for less than 50% obstruction, 2 for between 50-99% obstruction, and 3 for complete obstruction	
	2. Velum		
	3. Lateral pharyngeal walls and oropharynx		
	4. Tongue base		
	5. Epiglottis and Supraglottis		
Fishman et al. 2013 [13]	1. Nasal airway	Score of 0 for no obstruction, 1 for mild obstruction, 2 for moderate obstruction and 3 for severe obstruction	This system includes severity of OSA (mild, moderate and severe), level of confidence in findings, and quality of examination (poor, fair and good). The interpreter is asked to determine the primary site of obstruction or select a combination of sites
	2. Nasopharynx		
	3. Lateral pharyngeal walls and oropharynx		
	4. Tongue base		
	5. Epiglottis and supraglottis		
SERS [14]	1. Nasal airway	Score of 0 for no obstruction, 1 for partial obstruction, 2 for complete obstruction	
	2. Nasopharynx		
	3. Velum		
	4. Lateral pharyngeal walls and oropharynx		
	5. Hypopharynx		
	6. Larynx		
VOTE [15]	1. Velum	Score of 0 for no obstruction, 1 for partial obstruction, 2 for complete obstruction	This system characterizes the pattern of obstruction as anteroposterior, lateral or concentric
	2. Oropharynx and Lateral pharyngeal walls		
	3. Tongue base		
	4. Epiglottis		
Proposed Scoring System	1. Nasal airway	Airway patency is genearlly categrorized at each site to have no obstruction, partial obstruction, or significant obstruction	See Figure 1 for definitions of the obstructive categories at each site. A numeric scoring system could be applied to this system as needed for data collection with a score of 0 for no obstruction, 1 for partial obstruction, 2 for significant obstruction
	2. Adenoid		
	3. Palate		
	4. Tonsils and Lateral pharyngeal walls		
	5. Tongue base		
	6. Lingual tonsils		
	7. Vallecula		
	8. Epiglottis		
	9. Ayeroepiglottic folds		
	10. Arytenoids		

The VOTE system is the most studied scoring system and has been used in both children and adults [15]. The system is concise and easy to use. However, a major limitation of the VOTE scoring system for use in children is the omission of the nasopharyngeal and supraglottic sites. The Chan scoring system is similar to the VOTE system but includes the supraglottis and lingual tonsils specifically, which are important sources of obstruction in children [12,15]. The Chan system uses a scoring system which correlates to the percentage of obstruction at all sites except the lingual tonsil which is described only as present or absent [12]. The Bachar system and SERS are unique in that they are an overall score of upper airway obstruction [10,14]. The Boudewyns system is unique in that it includes a designation of generalized hypotonia as present or absent and characterizes whether the obstruction is fixed or dynamic [11]. The Fishman system evaluates the degree of obstruction at several upper airway subsites but it is unique in that it also notes the quality of the exam, confidence in the findings, and OSA severity [13]. While the validity and reliability of DISE is promising, the clinical significance of these scoring systems is not well established [5-7]. Our DISE scoring system attempts to evaluate all levels of potential airway obstruction while maintaining a simple and easy to follow guide. The ideal scoring system should be simple and practical with proven reliability and should completely characterize the nature of obstruction in order to guide management.

Another concern with DISE research is how accurately intravenous anesthesia simulates normal sleep for the patient. It is important to note that all anesthetics used for DISE have some documented effect on sleep architecture [5,16]. The anesthetic-specific effect on the degree or level of obstruction has not been well studied. A combination of dexmedetomidine and ketamine is preferred by some pediatric sleep surgeons due to the lower risk of respiratory depression and upper airway obstruction as seen with other agents [5,17]. In this protocol, propofol is used which has a more rapid onset and shorter duration as compared to dexmedetomidine. Propofol can result in deeper sedation and more pronounced airway collapse while dexmedetomidine provides longer lasting sedation with little effect on upper airway obstruction [16,18-20]. During DISE, it is critical that the depth of sedation is closely monitored in coordination with the anesthesiologist. There is an ongoing debate regarding the best anesthetic protocol for DISE and more research with direct comparison of these agents is needed. 

## Conclusions

The future clinical value of pediatric DISE depends on the continuation of ongoing research and development of future studies. At this time large-scale institutional studies, systematic reviews, and meta-analyses which could improve the power of DISE research are limited by the lack of a universally accepted technique and scoring system. Here, we have proposed a specific protocol for scoring of DISE in children. More research is needed to determine the reliability of scoring systems used for DISE and to determine the optimal anesthetic protocol used during DISE. 
